# Aged mouse ovaries possess rare premeiotic germ cells that can generate oocytes following transplantation into a young host environment

**DOI:** 10.18632/aging.100105

**Published:** 2009-12-12

**Authors:** Yuichi Niikura, Teruko Niikura, Jonathan L. Tilly

**Affiliations:** Vincent Center for Reproductive Biology, Vincent Obstetrics and Gynecology Service, Massachusetts General Hospital/Harvard Medical School, Boston, MA 02114, USA

**Keywords:** aging, germ cell, meiosis, Stra8, oogenesis, oocyte, ovary, stem cell

## Abstract

Of all the major organ systems in the body, the ovaries of females are the first to exhibit impaired function with advancing age. Until recently, traditional thinking was that female mammals are provided with a non-renewable pool of oocyte-containing follicles at birth that are depleted during postnatal life to exhaustion, driving ovarian failure. However, a growing body of evidence, including the isolation of germline stem cells (GSC) from adult mouse ovaries that produce developmentally-competent oocytes, has challenged this belief. In addition, rare germline stem-like cells capable of generating oocytes in vitro that undergo parthenogenesis to form blastocyst-like structures have recently been identified in postmenopausal human ovaries. Here we show that the germline-specific meiosis-commitment genes,Stimulated by retinoic acid gene 8 (Stra8) and Deleted in azoospermia-like (Dazl), are highly expressed in aged mouse ovaries. However, histological and marker analyses fail to demonstrate the presence of oocytes, supporting that Stra8 and Dazl are expressed in premeiotic germ cells that do not undergo further differentiation. Through the use of aged germline-specific GFP-expressing transgenic mice, we further show that these germ cells can generate GFP-positive oocytes that co-express the primordial oocyte marker NOBOX and form follicles when grafted into young adult wild-type female hosts. Thus, aged mouse ovaries possess a rare population of premeiotic germ cells that retain the capacity to form oocytes if exposed to a young host environment.

## Introduction

In humans and laboratory rodent models (rats and mice), the ovaries exhibit age-related dysfunction relatively early in life, with failure noted long before aging-associated changes in other organs are manifest. In humans, this loss of ovarian function drives the menopause and its associated increased risk for development of diverse health complications, many of which are tied to disrupted ovarian hormone production [1]. Endocrine function of the ovaries is carried out primarily by structures termed follicles, which are composed of a centralized germ cell arrested in meiosis (oocyte) surrounded by one or more layers of supporting somatic cells [2]. Traditional thinking has been that female mammals are provided with a non-renewable pool of oocyte-containing follicles at birth that are continuously depleted during postnatal life to the point of exhaustion [3]. However, a growing body of evidence (reviewed in [4]), including the recent purification and in-vitro propagation of premeiotic germ cells from neonatal and young adult mouse ovaries that can generate developmentally-competent oocytes in transplanted host females [5], has challenged this belief, thus offering new avenues to consider in the context of deciphering the role that adult stem cells may play in ovarian function and aging in females [6].

For example, findings from gene mutant mice show that p16^INK4a^ and p19^ARF^, two senescence-associated proteins that contribute to stem cell failure during aging of the hematopoietic, neural and cardiac systems [7-9], do not play a comparable role in restraining oogenesis in adult females [10]. However, another cell cycle-regulatory protein termed CABLES1 [cyclin-dependent kinase (CDK)-5 and ABL enzyme substrate 1] was identified as serving this function in the mouse female germline, uncovering a cell lineage-specificity with respect to the role that cell cycle modulators play in controlling somatic versus germline stem/progenitor cell activity [10]. Other studies have shown that postmenopausal human ovaries devoid of oocytes possess rare stem-like cells with germline characteristics [11]. When maintained in vitro under defined conditions, these cells spontaneously generate oocytes (or oocyte-like cells) that can undergo parthenogenetic develop-ment to form preimplantation embryo-like structures [12]. Although these reports indicate that aged ovarian tissue retains at least some degree of germline cell function, it is unclear whether these cells contribute to oogenesis under physiological conditions  and, if they do, why these cells would then fail to maintain the follicle reserve with advancing age. Herein we used mice as a model to further test whether changes in premeiotic germ cell function might be an important variable to at least consider in the context of understanding the mechanisms involved in ovarian aging in mammals.

**Figure 1.**
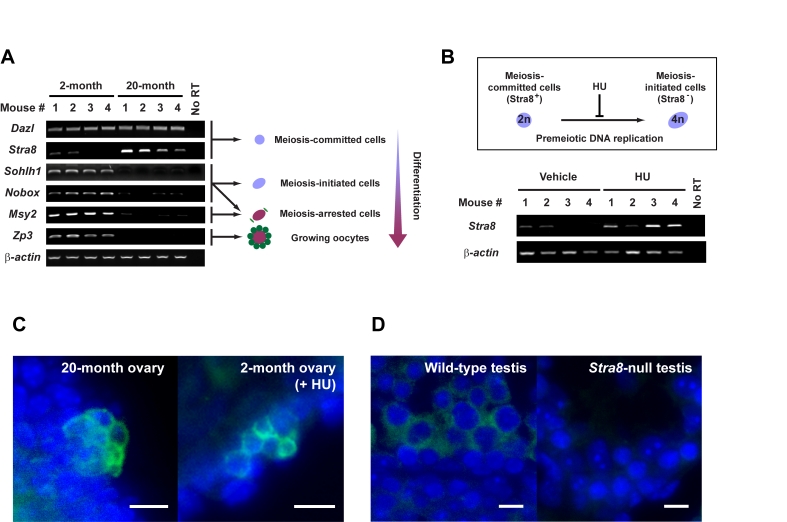
Premeiotic germ cells persist in aged atrophic mouse ovaries. (**A**) Analysis of germline marker gene expression in ovaries of young adult (2-month) and aged (20-month) female mice. Results from all 4 mice per age group are shown (*β-actin*, housekeeping gene used as a sample loading control). (**B**) In-vivo blockade of premeiotic DNA replication by HU in ovaries of young adult mice results in enhanced levels of *Stra8* expression, consistent with premeiotic germ cell accumulation. (**C**) Immunofluorescence analysis of STRA8 expression (*green*, cytoplasm) in ovaries of aged or HU-treated young adult female mice. (**D**) Control immunofluorescence analysis of STRA8 expression (*green, *cytoplasm) in testes of young adult wild-type or *Stra8*-null male mice (a representative cross-section of seminiferous tubule is shown for each.). C, D: scale bar = 10 μm; DAPI counterstain, *blue *(nucleus*)*.

## Results

### Premeiotic germ cells are present in aged atrophic mouse ovaries

To first determine if aged ovaries lacking oocytes possess premeiotic germ cells or stem cells with germline potential, we screened ovaries from young adult (2-month) and aged (20-month) C57BL/6 female mice for germline-specific gene expression. Genes associated with meiotic competence - namely *Deleted in azoospermia-like* (*Dazl*) and *Stimulated by retinoic acid gene 8* (*Stra8*) - were consistently detected in aged ovaries (Figure 1A). Complete oocyte depletion from ovaries of mice at these advanced ages was confirmed by both histological (data not shown) and gene marker analysis. Specifically for the latter, genes marking primordial oocyte formation (*Sohlh1*, *Nobox*) and diplotene-stage meiotically-arrested oocytes (*Msy2*) were variably (and very minimally) or not expressed; similarly, expression of a gene that marks growing oocytes (*Zp3*) was not detected in aged ovary tissue (Figure 1A). In contrast and as expected, young adult mouse ovaries which contain both premeiotic germ cells [5,13] and oocytes expressed all genes tested (Figure 1A). Cells expressing STRA8 protein, which heralds commitment of germ cells to meiotic entry by initiating premeiotic DNA synthesis [14], were localized to cells in the surface epithelium of aged ovaries, often detected in small cell clusters (Figure 1C). A similar pattern of STRA8 expression was observed in young adult mouse ovaries after in-vivo blockade of premeiotic DNA replication using hydroxyurea (HU) (Figure 1B and 1C). Thus, a rare population of premeiotic germ cells exists in atrophic ovaries of aged mice, but these cells are apparently unable to transition into oocytes.

**Figure 2.**
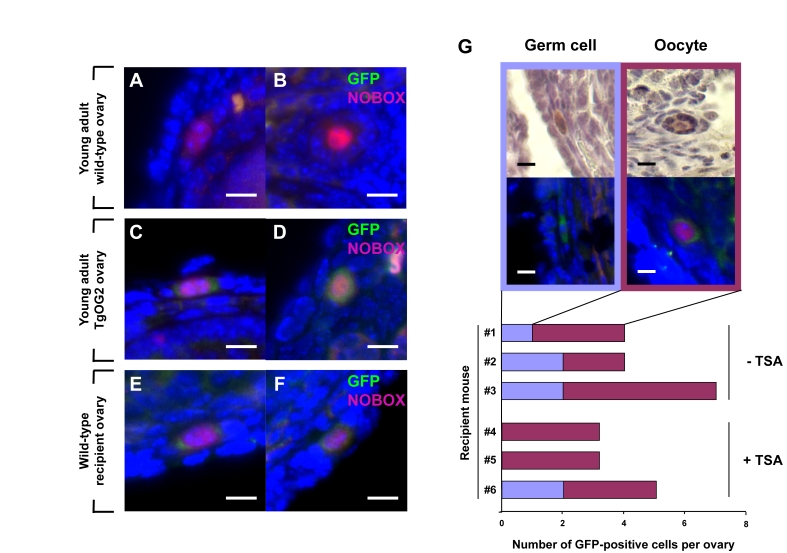
Young adult female mice support oocyte formation from germ cells in aged mouse ovaries. (**A-F**) Dual immunofluorescence analysis of GFP (*green*) and NOBOX (*red, *nucleus) expression in ovaries of young adult wild-type female mice (**A, B**), young adult TgOG2 female mice (**C, D**), and young adult wild-type recipient mice 6 weeks after proximal intrabursal grafting of aged TgOG2 ovarian tissue (**E, F**) (scale bar = 10 μm; DAPI, *blue, *nucleus). (**G**) Immunohistochemical detection of GFP (*brown*; upper), or dual immunofluorescence analysis of GFP (*green*) and NOBOX (*red, *nucleus) expression (middle; DAPI, *blue, *nucleus), along with numbers of non-follicle-enclosed GFP-positive germ cells and follicle-enclosed GFP-positive oocytes, in ovaries of wild-type young adult recipients treated with vehicle or TSA (recipient mouse #1-#3 or #4-#6, respectively) after proximal intrabursal grafting of aged TgOG2 ovarian tissue (scale bar = 10 μm).

### Dormant germ cells derived from aged ovaries can be re-activated to form oocytes

To test if these quiescent germ cells in aged mouse ovaries still possess the ability to generate oocytes and form follicles, we grafted ovarian tissue harvested from aged female germline-specific GFP-expressing mice (ΔPE-*Oct4*-*Gfp* or TgOG2 transgenic) into the ovarian bursal sacs of young adult wild-type female recipients. In brief, the bursal sac surrounding a wild-type host ovary was opened, and one-half of the host ovary was removed prior to inserting one-half of an ovary from an aged TgOG2 female in its place. The tissue was then allowed to settle back into the peritoneal cavity and the incision was closed. The remaining half of each aged TgOG2 ovary not transplanted was fixed immediately and processed for pre-grafting GFP expression analysis. Six weeks later, the mice were given a single intraperitoneal injection of vehicle or trichostatin-A (TSA), the latter of which enhances oogenesis in young adult and middle-age female mice [15,16]. Ovaries were collected 24 hours later for serial section immunohistochemical analysis of GFP-expressing cells. These experiments revealed an absence of GFP-positive germ cells in the aged ovarian tissue before grafting. However, a small number of GFP-positive germ cells, most of which were enclosed within somatic cells as immature follicles and co-expressed the primordial oocyte marker NOBOX [17], were detected after transplantation into a young host environment (Figure 2A-F). These germ cells and follicles were consistently observed in wild-type recipient ovaries close to the graft interface with aged transgenic donor ovary tissue, and the frequency of their detection was unaltered  by TSA exposure prior to collection (Figure 2G).

**Figure 3.**
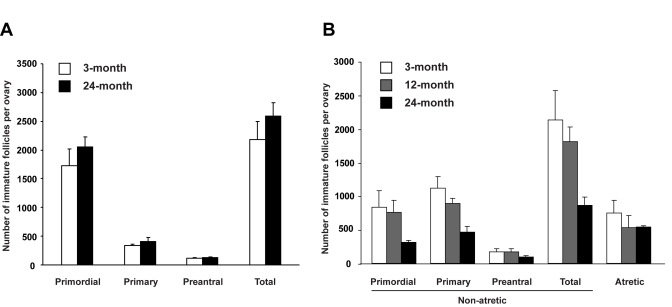
Assessment of the influence of age on the ovarian follicle reserve. (**A**) Immature follicle numbers in ovaries of young adult (2-month-old) female mice 7 weeks after parabiotic joining with either young adult (3-month-old) or aged (24-month-old) female mice (mean ± SEM, n = 5 mice per group). (**B**) Immature follicle numbers in young adult (2-month-old) mouse ovaries 3 weeks after grafting under the kidney capsules of young adult (3-month-old), middle-aged (12-month-old) or aged (24-month-old) female mice (mean ± SEM, n = 4 mice per group).

### Negative impact of age on maintenance of the follicle reserve

We next tested whether age-related systemic changes underlie the inability of aged ovaries to support oocyte formation from these rare premeiotic germ cells. To accomplish this, young adult female mice were parabiotically joined with young adult or aged female mice to examine if circulating factors from aged animals negatively affect the size of follicle reserve in ovaries of young adult females. Seven weeks after joining, comparable numbers of immature follicles were detected in ovaries of young adult mice joined with young adult or aged parabionts (Figure 3A). However, when young adult mouse ovaries were exposed directly to an aged systemic environment by grafting under the kidney capsules of 24-month-old female mice, immature follicle numbers in these ovaries were reduced within 3 weeks to less than 50% of those in age-matched ovaries grafted into young adult (3-month-old) or middle-aged (12-month-old) female recipients (Figure 3B). The change occurring between 12-24 months of age that triggers rapid deterioration of the follicle reserve may involve impaired oocyte renewal rather than accelerated loss, since the incidence of oocyte death (follicle atresia) was similar among groups (Figure 3B). This latter observation also indicates that loss of follicles from young ovaries grafted into aged recipients was not due to ischemia associated with reduced vascularization of the grafted tissue, since such an outcome would have been associated with elevated cell death.

## Discussion

Since initial claims that female mammals possess GSC and the ability to produce new oocytes and follicles during adult life [13], the existence of premeiotic germ cells and their potential roles in ovarian biology have been extensively debated (reviewed in [4,18]. However, skepticism surrounding this line work was greatly minimized by a recent study reporting on the purification of GSC (or at least their mitotically active progeny) from neonatal and young adult mouse ovaries [5,19]. These germ cells could not only be established and propagated in vitro for months, but were also shown to generate developmentally competent eggs that yielded viable offspring after transplantation into chemotherapy-conditioned adult female hosts [5]. By monitoring expression of *Stra8*, which is widely accepted as a germline-specific gene required for meiotic competency and commitment in mammals [14,20], herein we identified premeiotic germ cells in ovaries of aged mice that appear arrested in their ability to develop into oocytes.

**Figure 4.**
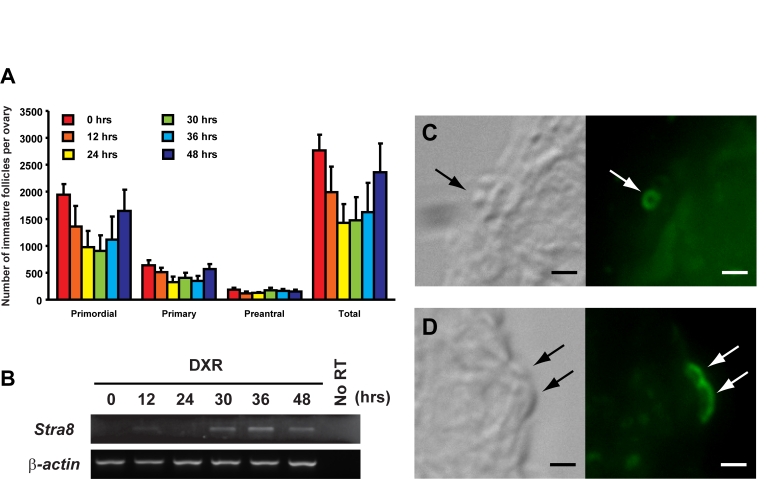
Induction of ovarian *Stra8* expression in adult female mice is correlated with oocyte renewal. (**A**) Number of non-atretic immature follicles in ovaries of 2-month-old mice at the indicated times following a single intraperitoneal injection of DXR (mean ± SEM, n = 4 mice per group). (**B**) RT-PCR analysis of *Stra8* expression in contralateral ovaries of 2-month-old mice at the indicated times following DXR injection (*β-actin*, house-keeping gene used as a sample loading control). (**C, D**) Examples of STRA8-immunopositive cells (*green*) in the surface epithelial layer of ovaries of mice 30 hours after DXR injection. Scale bar = 5 μm.

Interestingly, despite evidence for ongoing oocyte production in ovaries of young adult mice [13,15], (reviewed in [4]), STRA8-positive cells are rarely detected in young adult mouse ovaries [16]. This may reflect a quick transition of premeiotic germ cells, once committed by inducing *Stra8* expression, into oocytes during young adulthood.  Further, in young adult female mice the levels of detectable *Stra8* expression vary from ovary to ovary ([16]; present study), which is probably due to collection of ovaries from females without regard to stage of the reproductive cycle at the time of collection [16]. Indeed, past studies have shown that primordial follicle renewal in young adult female mice occurs only during metestrus and diestrus [15,21], and *Stra8* expression is more frequently detected in ovaries with lower than average oocyte counts presumably on the verge of estrous cycle-related replenishment [16]. In further support of this, STRA8-positive cells were consistently detected in young adult mouse ovaries after HU-mediated blockade of premeiotic DNA replication, which is an essential step for meiotic entry in mammalian germ cells. Thus, one would expect an accumulation of premeiotic (viz. *Stra8*-expressing) germ cells in the ovaries after HU treatment, similar to that reported to occur in the testes of HU-treated males [22]. Along these same lines, we previously demonstrated that immature follicles are rapidly regenerated in young adult mouse ovaries after acute oocyte loss induced by doxorubicin (DXR) exposure [15]. In the present study, we found a strong positive correlation between ovarian *Stra8* expression and regeneration of follicles following DXR treatment in young adult female mice (Figure 4). Similar to that observed in HU-treated young adult ovaries as well as in aged ovaries, STRA8-immunopositive cells were localized to the ovarian surface epithelium after DXR exposure, and were found at a time coincident with oocyte regeneration (Figure 4).

It is important to keep in mind, however, that the identification of premeiotic germ cells in aged ovary tissue - along with the demonstration that these cells retain the capacity to make oocytes if provided with a young host environment - should not be taken as evidence per se that these cells play a key role in the normal biology of the ovary or in the loss of its function with age. Nevertheless, these observations at least open the possibility that ovarian failure in mammals reflects not just atretic depletion of the oocyte pool to the point of exhaustion but also a progressive loss of oocyte input back into the ovarian reserve due to impaired function of premeiotic germ cells that are capable of generating new oocytes. Whatever the case, it bears mention that atrophied testes of aged male mice which no longer produce sperm are known to retain quiescent GSC capable of driving spermatogenesis in a young host environment following transplantation [23,24]. Thus, our new findings presented herein, along with recent data raising serious questions over the long-held belief that the mammalian ovary is endowed with a non-renewable oocyte pool at birth [4,5,13,15,16], provide an impetus to further explore if aging-related gonadal failure might occur through similar stem cell failure-based mechanisms in males and females.

## Methods

Animals and reagents. Wild-type C57BL/6 female mice were obtained from Charles River Laboratories (Wilmington, MA) or the National Institute on Aging (Bethesda, MD). Transgenic mice with GFP expression driven by a modified *Oct-4* promoter (ΔPE-*Oct4*-*Gfp* or TgOG2 mice) to convey germline specificity of the transgene [4,25-28] were obtained from J.R. Mann through K.J. MacLaughlin (University of Pennsylvania, Kennett Square, PA). For blockade of premeiotic DNA replication, 2-month-old wild-type female mice were given intraperitoneal injections of HU (Sigma Chemical Co., St. Louis, MO) (500 mg/kg body weight, in saline; 100 μl total volume per injection) every 12 h for 2-3 days [22]. Doxorubicin (Sigma) was administered as a single intraperitoneal injection (5 mg/kg body weight, in saline; 100 μl total volume). Trichostatin-A (Sigma) was also administered as a single intraperitoneal injection (10 mg/kg body weight, in DMSO; 100 μl total volume). The institutional animal care and use committee of Massachusetts General Hospital reviewed and approved all animal procedures described herein.

RT-PCR analysis. Total RNA was extracted from each whole ovary sample followed by DNase treatment to eliminate contaminating genomic DNA, and 1 μg was reverse transcribed (Superscript II RT; Invitrogen, Carlsbad, CA) using oligo-dT primers. Amplification via 26-45 cycles of PCR was then performed using platinum *Taq* polymerase (Invitrogen) and Buffer-D (Epicentre, Madison, WI). For each sample, mRNA encoded by the *β-actin* gene was amplified and used as a sample loading control for standardization. All PCR products were subcloned and sequenced for confirmation. Forward and reverse primers used were as follows, with GenBank Accession Number, size of amplified cDNA product and region of coding sequence amplified indicated:

**Table:** 

*β-actin* (Accession No. X03672; 439-bp product, nucleotides 4-443)
5'-GATGACGATATCGCTGCGCTG-3'
5'-GTACGACCAGAGGCATACAGG-3'
*Dazl* (Accession No. NM_010021; 317-bp product, nucleotides 230-547)
5'-GTGTGTCGAAGGGCTATGGAT-3'
5'-ACAGGCAGCTGATATCCAGTG-3'
*Msy2 *(Accession No. NP_058571; 637-bp product, nucleotides 676-1313)
5'-CCACCACCCTTCTTCTATCGA-3'
5'-GGTGATGCCTCGGAACAATA-3'
*Nobox *(Accession No. AY061761; 378-bp product, nucleotides 1088-1466)
5'-CCCTTCAGTCACAGTTTCCGT-3'
5'-GTCTCTACTCTAGTGCCTTCG-3'
*Sohlh1 *(Accession No. NP_001001714; 234-bp product, nucleotides 288-522)
5'-GATGTCTGTGTACTTCCTCC-3'
5'-CTGGCTCACTGAATGACAAC-3'
*Stra8* (Accession No. NP_033318; 631-bp product, nucleotides 429-1060)
5'-GCCAGAATGTATTCCGAGAA-3'
5'-CTCACTCTTGTCCAGGAAAC-3'
*Zp3 *(Accession No. M20026; 182-bp product, nucleotides 50-232)
5'-CCGAGCTGTGCAATTCCCAGA-3'
5'-AACCCTCTGAGCCAAGGGTGA-3'

Oocyte (follicle) counts. Ovaries were fixed in a solution containing 0.34 N glacial acetic acid, 10% formalin and 28% ethanol, and embedded in paraffin. Serial sections were cut (8 μm), aligned in order on glass slides, and stained with hematoxylin and picric methyl blue. The number of non-atretic and atretic immature (primordial, primary, and preantral) follicles per ovary was then determined as detailed previously [29,30].

Immunodetection of STRA8, GFP and NOBOX. Ovaries were fixed in 4% neutral-buffered paraformaldehyde at room temperature for 3-4 hours and embedded in paraffin. Tissue sections (6 μm) were cut and mounted on glass microscope slides. Sections were de-waxed in xylenes, re-hydrated in a graded ethanol series, and then boiled in 10 mM sodium citrate for antigen retrieval [15,16,31]. A chicken polyclonal anti-STRA8 antibody was generated in chickens (Aves Labs, Tigard, OR) using the synthetic peptide, QEQEESLDKLLKLKAS, which corresponds to amino acids 76-91 of the mouse STRA8 coding sequence. For fluorescence visualization, a biotin-conjugated goat-anti-chicken IgY (B-1005; Aves Labs) and a streptavidin-conjugated Alexa Fluor-488 (S11223; Molecular Probes, Eugene, OR) were used. For chromogenic visualization of GFP expression, a mouse monoclonal antibody against GFP (sc-9996; Santa Cruz Biotechnology, Santa Cruz, CA) was used in conjunction with the MOM Kit (PK2200; Vector Labs, Burlingame, CA) for antigen detection [15,28]. For dual immunofluorescence analysis of GFP and NOBOX expression [15], GFP detection was first performed using a mouse monoclonal antibody against GFP along with the MOM Kit (see above) and a streptavidin-conjugated Alexa Fluor-488 probe (Molecular Probes) followed by NOBOX staining using a rabbit polyclonal anti-NOBOX antibody (ab41521; Abcam, Cambridge, MA) with Alexa Fluor-568 conjugated goat anti-rabbit IgG (A11011; Invitrogen). Sections were mounted with DAPI (Vectashield; Vector Labs), and images were captured using a Nikon ECLIPSE TE2000-S microscope equipped with an EXFO X-Cite 120 fluorescence illuminator. Positive and negative controls, consisting of ovarian sections from young adult TgOG2 and wild-type females, respectively, were always included with the experimental tissues on each slide.

Parabiosis. Each parabiont (young adult wild-type female mouse joined with either a young or aged wild-type female mouse) was anesthetized and an incision was made from the olecranon to the knee joint of each mouse. The olecranon and knee joint were attached by a single 5-0 Polyglactin 910 suture and tie, and the dorsal and ventral skin flaps were approximated by staples and suture [32]. Seven weeks after surgery, ovarian tissues were collected and processed for follicle counts.

Ovarian grafting. For kidney capsule transplants, young (3-month-old), middle-age (12-month-old) or aged (24-month-old) wild-type female mice were anaesthetized (Avertin, 200 mg/kg, intraperitoneal) to expose the left kidney in each mouse through a dorso-lateral incision. For each recipient animal, a small space was made under the kidney capsule, and ovaries collected from young adult (2-month-old) wild-type donor mice were placed into the space. At the same time, both host ovaries were removed. The kidney was then allowed to settle back into the peritoneal cavity and the incision was closed. Three weeks after surgery, grafted ovaries were removed from host kidneys and processed for follicle counts. For grafting into ovarian bursal sacs, similar surgical procedures were followed with the exception that the left ovary instead of the kidney of each recipient mouse was exposed. The bursal sac surrounding the ovary was opened, and one-half of the wild-type host ovary was removed prior to inserting one-half of an ovary from an aged TgOG2 female in its place, essentially as described [13]. The tissue was then allowed to settle back into the peritoneal cavity and the incision was closed. To help facilitate oogenesis some of the wild-type recipient female mice were given a single intraperitoneal injection of TSA 6 weeks after surgery [15,16]. Twenty-four hours later, the grafted ovaries were removed and serially-sectioned for analysis. The remaining half of each aged TgOG2 ovary not transplanted was fixed immediately and processed for pre-grafting GFP expression analysis.

Data presentation and analysis. All experiments were independently replicated at least 3 times (see figure legends for details), using different mice in each experiment. Where appropriate, assignment of mice to each experimental group was made randomly. Quantitative data from experimental replicates were pooled and presented as the mean ± SEM. Representative outcomes from the RT-PCR and immunodetection analyses are provided for qualitative assessment.
